# A cerebellum inspired spiking neural network as a multi-model for pattern classification and robotic trajectory prediction

**DOI:** 10.3389/fnins.2022.909146

**Published:** 2022-11-28

**Authors:** Asha Vijayan, Shyam Diwakar

**Affiliations:** ^1^Amrita Mind Brain Center, Amrita Vishwa Vidyapeetham, Kollam, India; ^2^School of Biotechnology, Amrita Vishwa Vidyapeetham, Kollam, India; ^3^Department of Electronics and Communication, Amrita School of Engineering, Amrita Vishwa Vidyapeetham, Kollam, India

**Keywords:** cerebellum, spiking neural network, deep learning, encoding, classification, trajectory prediction

## Abstract

Spiking neural networks were introduced to understand spatiotemporal information processing in neurons and have found their application in pattern encoding, data discrimination, and classification. Bioinspired network architectures are considered for event-driven tasks, and scientists have looked at different theories based on the architecture and functioning. Motor tasks, for example, have networks inspired by cerebellar architecture where the granular layer recodes sparse representations of the mossy fiber (MF) inputs and has more roles in motor learning. Using abstractions from cerebellar connections and learning rules of deep learning network (DLN), patterns were discriminated within datasets, and the same algorithm was used for trajectory optimization. In the current work, a cerebellum-inspired spiking neural network with dynamics of cerebellar neurons and learning mechanisms attributed to the granular layer, Purkinje cell (PC) layer, and cerebellar nuclei interconnected by excitatory and inhibitory synapses was implemented. The model’s pattern discrimination capability was tested for two tasks on standard machine learning (ML) datasets and on following a trajectory of a low-cost sensor-free robotic articulator. Tuned for supervised learning, the pattern classification capability of the cerebellum-inspired network algorithm has produced more generalized models than data-specific precision models on smaller training datasets. The model showed an accuracy of 72%, which was comparable to standard ML algorithms, such as MLP (78%), Dl4jMlpClassifier (64%), RBFNetwork (71.4%), and libSVM-linear (85.7%). The cerebellar model increased the network’s capability and decreased storage, augmenting faster computations. Additionally, the network model could also implicitly reconstruct the trajectory of a 6-degree of freedom (DOF) robotic arm with a low error rate by reconstructing the kinematic parameters. The variability between the actual and predicted trajectory points was noted to be ± 3 cm (while moving to a position in a cuboid space of 25 × 30 × 40 cm). Although a few known learning rules were implemented among known types of plasticity in the cerebellum, the network model showed a generalized processing capability for a range of signals, modulating the data through the interconnected neural populations. In addition to potential use on sensor-free or feed-forward based controllers for robotic arms and as a generalized pattern classification algorithm, this model adds implications to motor learning theory.

## Introduction

The brain circuits of many animals have significantly improved their capacity to learn and process multimodal inputs (Stock et al., 2017) at the millisecond scale that machine learning (ML) algorithms have abstracted to classify (Albus, 1975) or cluster data (Kohonen, 1982). Yet, these algorithms are not as complex or efficient as the brain’s neural circuits (Luo et al., 2019). Modern methods, such as deep learning networks (DLN) and extending artificial neural networks (ANN), may bring additional similarities to the computational capabilities of neural circuits while predicting and classifying patterns within big and small datasets (Angermueller et al., 2016), which could also help models to learn and think like humans (Lake et al., 2017). Studies involving DLN models suggest its application in deducing information processing within biological networks (Yamins and DiCarlo, 2016), in addition to disease-related predictions attributed to impaired network activity (Yang et al., 2014). Many DLN models have been inspired by the brain’s microcircuit architectures, such as the visual cortex (Kindel et al., 2019), basal ganglia (Hajj and Awad, 2018), and hippocampus (Fontana, 2017), and these neuro-inspired models explore novel functional relationships within data.

Spiking neural networks (SNN) exploit a biologically observed phenomenological element in ML, allowing optimization and parallelizability to algorithms (Naveros et al., 2017) which may be event-driven and time-driven and may incorporate spatiotemporal information processing capabilities of biological neural circuits. Algorithms that are based on different brain circuits, such as the visual cortex (Fu et al., 2012; Yamins and DiCarlo, 2016), basal ganglia (Doya, 2000; Baladron and Hamker, 2015; Girard et al., 2020), and cerebellum (Casellato et al., 2012; Garrido et al., 2013; Antonietti et al., 2015; D’Angelo et al., 2016b; Luque et al., 2016; Yamaura et al., 2020; Kuriyama et al., 2021) with spiking neural models help understand the circuitry and in turn, help reconstruct and train systems. EDLUT (Ros et al., 2006), SpiNNaker (Khan et al., 2008), MuSpiNN (Ghosh-Dastidar and Adeli, 2009), and biCNN (Pinzon-Morales and Hirata, 2013) are some of the existing brain-inspired models which are used in the field of control systems for robotic articulation control. In this article, we mathematically reconstructed a cerebellum-inspired neural circuit incorporating the training efficacy of a deep learning classifier for applications in motor articulation control and pattern classification.

**FIGURE 1:**
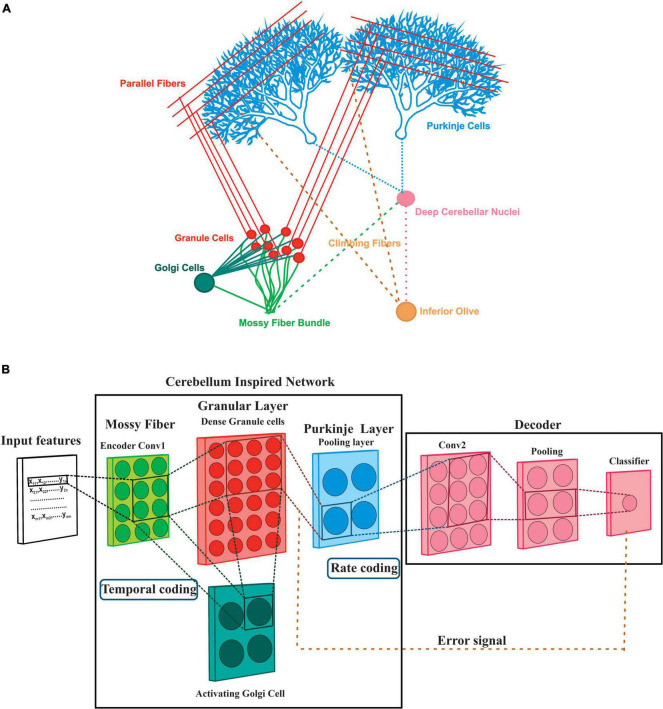
A deep layer network of the cerebellum. **(A)** The abstracted model was based on the simplified cerebellar microcircuitry of rat cerebellum: Various neurons and connections in the cerebellar circuitry may attribute to pattern classification and prediction. Input patterns are presented through the Mossy fibers (MF) which have projections on the Granule cells (Grc) extended as Parallel fibers (PF) and Golgi cells (GoC). PF impinges onto the Purkinje cells (PC) which gives input to the output neuron Deep Cerebellar Nuclei (DCN) which in turn evokes Inferior Olive (IO). From a deep learner perspective, the cerebellum has been known to perform supervised learning with the teaching signals send to the PC through the IO extension Climbing fibers (CF). **(B)** The equivalent model of deep learning involved a spiking neural network: All the neurons inside the cerebellum-inspired network were reconstructed as spiking models whereas the decoder was modeled with nodes. Each instance was mapped to a convolution layer that encodes to a group of MF. Convoluted cells mapped to a dense granular layer (Diwakar et al., 2011) representing the sparseness of the MF stimuli which is also activated by an inhibitory GoC model. Rate coding of the inputs, convolution at the GrC layer, and SoftMax based output prediction post the pooled layer was implemented at the MF-GrC-PC circuit.

Among the different brain regions, the cerebellum, situated inferior to the occipital lobe of the cerebral cortex, has a modular structure and shares a common architecture (Standring, 2016) known to be involved with learning at different layers. Cerebellum follows a well-organized network structure (Figure 1A) with afferent circuitry involving two primary excitatory inputs to the cerebellum originating through the mossy fibers (MF) and climbing fibers (CF). Sensory and tactile inputs from different regions, including the brain stem and the spinal cord, are transferred by MF as input signals. At the same time, CF has been known to provide training errors compared with inputs from the inferior olive (IO) (Mauk et al., 1986). IO receives input from deep cerebellar nuclei (DCN), which are inhibited by the Purkinje neurons and considered to be the main output neurons of the cerebellar cortex (Eccles, 1973; D’Angelo et al., 2016a). The cerebellum granular layer has a large number of granule cells (GrC), significantly lesser numbers of Golgi cells (GoC), and a few Unipolar Brush Cells (UBC), while the primary neurons of the molecular layer are the Purkinje cells (PC). Due to the error correction mechanism by the IO onto the PC, the cerebellum has been known to perform a supervised motor learning of sensory and movement patterns (Porrill et al., 2004; Passot et al., 2010), although the relationship between the cerebellum and motor learning was suggested even earlier (Cajal, 1911). On the other hand, GrCs have been known to involve in sparse recoding of the different sensory modalities. The implementation of inhibition-based looping in the network model suggests that feed-forward inhibition of GrC may be crucial in modulating the efficacy of pattern discrimination of the PC, which is attributed to some of the biophysical characteristics of the PF-PC synapses. Generalization and learning-induced accuracy (Clopath et al., 2012) in the network model involves clustered activation (Diwakar et al., 2011) and synchronous behavior (Maex and De Schutter, 1998) in the GrC.

Sensorimotor control in the brain is often attributed to the cerebellum (Kawato and Gomi, 1992; Schweighofer et al., 1998; Thach, 1998; Ito, 2002; Nowak et al., 2007; Haith and Vijayakumar, 2009; Popa et al., 2012), which has been known to play roles in the error correction based control (Tseng et al., 2007; Shadmehr et al., 2010; Luque et al., 2011; Popa et al., 2016) by timing and coordinating movement (D’Angelo and Zeeuw, 2008). Studies have shown that the brain-inspired NN with a feed-forward and feedback controller could control a visually guided robotic arm (Schweighofer and Arbib, 1998; Tseng et al., 2007), saccades (Schweighofer et al., 1996), smooth pursuit (Kettner et al., 1997), and eye blinking condition (Casellato et al., 2014). Multiple controllers have several copies of inverse and forward models that could be coupled together to attain the task of fast and distributed coordination (Wolpert and Kawato, 1998; Kawato, 1999) and have been mainly used in unsupervised (Schweighofer et al., 2001) and supervised learning (Kawato et al., 2011) but not have had much focus on reinforcement learning (Yamazaki and Lennon, 2019; Kawato et al., 2020). Real-time processing of information for error detection and correction (Rodriguez-Fornells et al., 2002; Yamazaki and Igarashi, 2013; Luo et al., 2016; Popa et al., 2016; Yamazaki et al., 2019), predictive control (Kettner et al., 1997), the timing of muscle synergies (Manto et al., 2012), and formation of an internal model for supervised learning (Doya, 1999) as well as for calculating inverse dynamics (Kawato and Gomi, 1992) was attributed to the cerebellum because of their multi-faceted role in motor learning (Tanaka et al., 2020).

The cerebellum has similarities to DLN models with different learning modules and known supervised learning attributions (Eccles et al., 1967b; Marr, 1969; Albus, 1971; Ito, 1972; Doya, 2000). The memories formed can be stored in the cerebellar cortex and the deep nuclei. At the same time, the cerebellum elaborates over 16 known learning mechanisms (Mapelli et al., 2015), and the cerebellar cortex has been experimentally observed to be critical for regulating the timing of movements and learning is transferred partially or wholly to the deep nuclei. Plasticity at the MF-GrC connection (Nieus et al., 2006) has been less explored when compared with PF-PC plasticity. Olivo-cerebellar tract has also been seen to have a crucial role in learning new motor skills (Ito, 2006). Other hypotheses include the cerebellum as an adaptive filter (Fujita, 1982), a motor pattern classifier (Albus, 1975), and a neuronal timing machine that adapts to motor tasks (D’Angelo and Zeeuw, 2008). Cerebellum-inspired models have also been used in robotics, pattern separation, and ML applications (Ros et al., 2006; Dean et al., 2010; Tanaka et al., 2010; Casellato et al., 2014; Antonietti et al., 2015). Cerebellum-inspired network models have been attributed to several functions, such as vestibulo-ocular reflexes (VOR), optokinetic responses (OKR) (Inagaki and Hirata, 2017), eye blink classical conditioning (EBCC), and motor control with these different mechanisms attributed to the same neural circuitry relating a deep learning aspect into the cerebellum microcircuits (Wolpert et al., 1998; Hausknecht et al., 2016).

Sensory modalities evoke sparse activation of neurons where some neurons remain active all the time, whereas other neurons respond to few stimuli (Pehlevan and Sompolinsky, 2014) and this behavior has been observed in different parts of the brain, such as the cortex and cerebellum. Early studies (Marr, 1969; Albus, 1971) have suggested the sparse role of cerebellar granular layer neurons improves pattern separation. A measure of the sparseness or storage capacity of neurons was used to understand the responses of the network (Brunel et al., 2004; Pehlevan and Sompolinsky, 2014) at different sensory stimuli conditions in areas, such as the sensory cortex (Fiete et al., 2004), cerebellum (Clopath and Brunel, 2013; Babadi and Sompolinsky, 2014; Billings et al., 2014), etc. Models developed based on the cerebellum may be crucial for simulating movement changes in related disorders, such as spinocerebellar ataxia (Sausbier et al., 2004; Kemp et al., 2016) and multiple sclerosis (Redondo et al., 2015; Wilkins, 2017). As sparseness and capacity indicate the amount of computation and storage, these measures are now being used in modeling. They are also calculated in terms of bits of information processed (Varshney et al., 2006) which may be employed to assess DLNs. Here, we reconstructed a cerebellum-inspired spiking neural network with aspects of biophysical dynamics and some of the learning mechanisms of the cerebellum. The focus of this article was also to build a multipurpose algorithm that could perform tasks, such as pattern encoding, discrimination, separation, classification, and prediction critical to motor articulation control. The modeled network demonstrates capabilities attributed to a pattern classifier and functions implicitly as a trajectory predictor for motor articulation control resembling the sensorimotor control observed in the cerebellum. The simulated cerebellar network was analyzed for its generalization capability aiming for pattern recognition and supervised learning. Toward quantifying performance, the model implemented on CPUs was compared with implementations on graphics processing unit (GPUs) and against some of the other learning algorithms.

In the current study, we aim to reconstruct a cerebellum-inspired spiking neural network extended from a previous study (Vijayan et al., 2017) that could perform tasks with multiple configurations like pattern classification and trajectory prediction using the same network architecture. The network has been mathematically reconstructed for two aspects: an extension of the theory of cerebellar function (Marr, 1969; Albus, 1971; Ito, 1982) where we looked at employing the modern understanding of the cerebellar cortex and at the same time, develop a neuro-inspired bio-realistic approach for low-cost robotic control. Unlike in the previous model (Vijayan et al., 2017), which had a granular layer with only GrCs, we have incorporated the recoding and associative mapping properties of the cerebellar granular layer with excitatory GrC and an inhibitory GoC, pattern discrimination at the Purkinje layer, and the interpretational application of DCN. Moreover, the network size was scalable. Extending the Marr–Albus–Ito theory, we have included learning in the granular layer, suggesting there is a whole set of operations that are done by the granular layer and would be performed as indicated by some experimental studies (D’Angelo et al., 2001, 2013; Rössert et al., 2015). The reconstructed cerebellum-inspired spiking neural network model performed tasks of different mathematical capabilities of this connectivity and circuit and can be repurposed to help model disease conditions as well as in developing controller models for sensor-free neuroprosthesis.

## Materials and methods

### Neuronal models for spiking networks

A cerebellum-inspired spiking neural network model (Figure 1B) with deep learning functionality was developed, extending our previous implementation (Vijayan et al., 2017). The input stimuli (MF) and the cerebellar neurons, such as GrC, GoC, and PC, were reconstructed using the Adaptive Exponential Integrate-and-Fire (AdEx) model (Naud et al., 2008). The nine parameter values for the AdEx model were obtained using a PSO algorithm (Table 1). The firing behavior was reconstructed for a single neuron with specific current values (Table 1) and matched the experimental recordings from p17 to 23 rat cerebellum cerebellar neurons (D’Angelo et al., 2001) and validated with modeling articles (Medini et al., 2012, 2014). Spiking dynamics and the neuron’s firing rate adaptation were modeled using Eqs (1)–(3).

**TABLE 1:** Parameter values used in modeling the different types of neurons.

Parameter	Values		
	GrC	GoC	PC
C (pF)	150	500	100
g_L_ (nS)	10	13.1	10
E_l_ (mV)	−70	−58	−65
Δ_T_ (mV)	4	7	2
V_t_ (mV)	−50	−54	−50
τ_w_ (mS)	13	8.7	1
I (pA)	350	0	0
a (nS)	9	−17	−13
b (pA)	250	1,033	260


(1) $$\text{C}\frac{d\text{V}}{d\text{t}}=-g_{L}\left(V-E_{L}\right)+g_{L}{△}_{T}\exp\left(\frac{V-V_{T}}{△T}\right)+I-w$$

(2) $$\tau_{w}\frac{dw}{dt}=\text{a}\left(V-E_{L}\right)$$

(3) $$IfV>0mVthen\left\{ \begin{array}{c} V=V_{r}\\ w=w+b \end{array}\right.$$

Here, C was the membrane capacitance, g_*L*_ represented leak conductance, E_*L*_ denoted resting potential, Δ_*T*_ represented slope factor, and *V_T_* denoted threshold potential. Variable “w” described the adaptation factor within the membrane potential, and “a” represented the relevance of sub-threshold adaptation. “I” referred to the injected current applied from an external source, and “b” referred to the spike-triggered adaptation constant.

**FIGURE 2:**
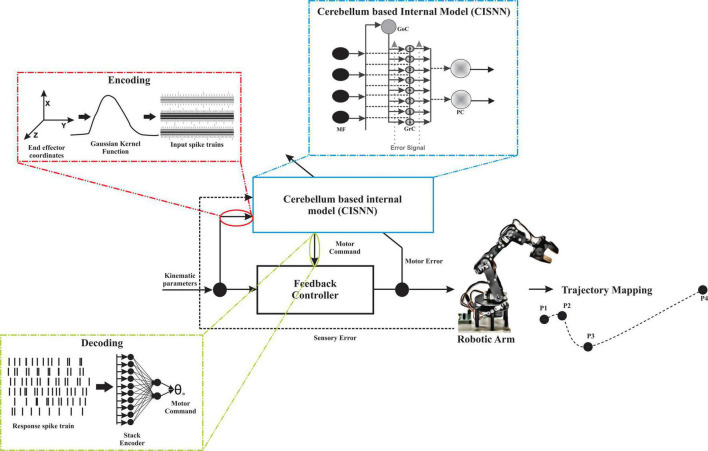
The network topology for robotic abstraction to predict trajectory. Input parameters (kinematic parameters) were encoded to MF spike times using a Gaussian kernel and are temporal coded. The temporal coded information is given as input to the cerebellum-inspired spiking neural network (CISNN). CISNN is a layered network with MF, GrC, and PC layers. The output of CISNN was obtained as PC spike times which were then rate coded by employing a stack encoder, the output obtained was presented to a low-cost robotic articulator that mapped the 4-point trajectory. Changes in sensory and motor values were calculated and fed back to the network to learn and update the existing weights.

The cerebellum-inspired deep learning algorithm included four component modules: an encoder to translate real-world data, a spiking cerebellar microcircuit-based network, a decoder of spiking information, and a learning and adaptation module to update weights at different layers in the algorithm (Figure 2).

#### Encoding of real-world data

Dataset^1^ used as training input consisted of different features [x_1_,x_2_,x_3_…x_*n*_] ∈ X with a class label of y. The input features were encoded (Algorithm 1) into spikes using a convoluted Gaussian kernel function. The attributes were mapped to a dataset-based variable in higher dimensional space, simulating the MF-GrC elaboration. Model currents were estimated as weighted input values while computing spikes from the encoded data through the granular layer. In the mapping of inputs, a normal distribution similar to postsynaptic latencies (Silver et al., 1996) allowed a center-surround structure (Mapelli et al., 2010) to the inputs (neuron in the center received the strongest excitation). The neurons were made user-defined and scalable. The convolution layer in DLN was designed to abstract the relevant features from a dataset. In the cerebellum, the MF have been known to bring relevant information from the higher centers (Figure 1).

**Algorithm 1:** MF-granular input encoding using a Gaussian Kernel based convolution.

1. Preprocess data **X** by normalizing the features using **Min-Max normalization** $D_{norm}=\frac{x_{i}-\text{min}\left(x\right)}{\max\left(x\right)-\text{min}\left(x\right)}*\left(n_{max}-n_{min}\right)+n_{min}$ Where X is the dataset [x_*i*_] ∈ X, *n*_*max*_ is the new maximum, *n*_*min*_ is the new minimum 2. For each instance ***i*** 3. For each attribute ***j*** 4. Compute **f**(**D_norm_**(**i, j**)) pdf for using a **normal distribution function** 5. Compute convolution matrix ***C*** by using a full convolution of the pdf obtained and **D_norm_**(**i, j**). 6. Compute the scalable datapoint for each feature ***D_*j*_* = [μ*_*ij*_-3σ***, **μ_*ij*_-2σ, μ_*ij*_-σ, μ_*ij*_, μ_*ij*_+σ, μ_*ij*_+2σ**, **μ*_*ij*_+3σ*]** 7. Compute ***w*_j_** which was **f**(μ_**i***j*_) 8. Check if **C** contains datapoints 9. Compute I = w_j_*μ_**i***j*_ 10. End if 11. End for 12. For each **D** 13. Create spiking neurons with the computed **I** as current 14. End for 15. End for

#### Cerebellum-inspired spiking neural network

A scalable cerebellar network was modeled (Algorithm 2) consisting of MF, GrC, GoC, and PC models. One of the aspects of reconstructing a cerebellar network was to employ the modern understanding of the cerebellar cortex and to solve motor task classification and prediction. Each feature was represented as a set of *n* neurons. The number of MF inputs was set based on the dataset feature size. For a dataset with 4 features and with 7 neurons per feature (see Supplementary Method 5 and Supplementary Table 1), the network consisted of 28 MFs, 371 GrCs, 1 GoC, and 1 PC. Twenty-eight MFs provided excitatory input to 371 GrCs and 1 GoC. An inhibitory GoC connection modulated all GrCs and its projection on the PC was considered as the output to the network. Convergence was set as in previous studies (Solinas et al., 2010; Vijayan et al., 2017). The weights were initialized using the standard approach of randomly picking from a normal distribution at each layer to avoid the explosion or vanishing of the activation layer output. The model was simulated for 300 ms. Each MF was mapped to 53 granule cells considering the convergence–divergence ratio (Eccles et al., 1967a). Every feature was mapped to 7 neurons, so for a 4-featured data point, there were 28 input neurons (MF) mapped to GrC and then to the PC. The output of the PC was decoded to the final output through 3 layered networks. The Purkinje layer was considered as the pooling layer where the output from the dense granular layer was summed up. The output from the pooled layer was sent to the second convolution layer, where the features are extracted, pooled, and classified (Figure 1B).

**Algorithm 2:** Cerebellum inspired spiking neural network.

1. Create **n** MF neurons with ***x*_*j :*_N_MF_** = n*x_j_ 2. Create Granular layer 3. Golgi cells **GoC** created based on the MF count **N_MF_** 4. Connection weights **w_MF–GoC_** were set to a random range of values [0.01–0.09] of size **N_MF_ x N_GoC_** Where **N_GoC_** is the number of Golgi cells 5. For each **GoC** 6. For each **MF** 7. Compute ***I*_***GoC***_** by adding **w_MF–GoC_** **(MF, GoC)** at time **T_MF_** Where **T_MF_** is the Mossy fiber spike time 8. End for 9. Create Golgi neuron models with the computed ***I*_***GoC***_** using Eq. (1)-(3) 10. End for 11. Compute $N_{\mathrm{GrC}}=\frac{\left(N_{\mathrm{MF}}*53\right)}{4}$ 12. Granule cells **GrC** created based on **N_MF_, T_MF_, N_GoC_, T_GoC_** Where **T_GoC_** is the Golgi cell spike timings 13. Connection weights **w_MF–GrC_** were set to a uniform random value of size **N_MF_ x N_GrC_** 14. Connection weights **w_GoC–GrC_** were set to a random range of values [0.01–0.09] of size **N_GoC_ x N_GrC_** were set 15. Number of activated MF, **A_MF_**, for each **GrC** was set to 4 16. For each **GrC** 17. For each **A_MF_** 18. Compute ***I_***GrC***_ = I_***GrC***_*+ w_MF–GrC_** **(A_MF_,GrC)** at **T_MF_** 19. End for 20. For each **GoC** 21. Compute ***I*_***GrC***_ = I_***GrC***_- w_GoC–GrC_** **(GoC, GrC)** at Golgi cell spike time **T_GoC_** 22. End for 23. Create Granule neuron model with the computed **I_GrC_** and the spike train using Eq. (4) 24. End for 25. Purkinje cells **PC** was created based on the number of output features **y_j_;** **N_PC_ = count (y_j_)** 26. Connection weights **w_GrC–PC_** were set to a value of 0.01 of size **N_GrC_ x N_PC_** 27. Number of activated GrC, **A_Grc_**, for each **PC** was set as 48 28. For each **PC** 29. For each **A_GrC_** 30. Compute ***I***_*PC*_ = **f**(***T***_*GrC*_) * **w_GrC–PC_ (A_GrC_,PC)** and the spike train using Eq. (7) 31. End for 32. Create Purkinje neuron model with the computed **I_PC_** using Eq. (1)-(3) 33. End for

The spike trains for each neuron in the granule layer was defined by Eq. (4)


(4) $$PSP_{Grc}=w^{T}x_{MF}\left(t\right)=\sum w_{MF}x_{MF}\left(t\right)$$

Where *w^T^* was the complete weight matrix for the MF-GrC connection in which *x*_*MF*_ was calculated using Eq. (5)


(5) $$x_{MF}\left(t\right)=\sum_{{\text{t}}^{f}<t}\delta \left(t-{\text{t}}^{f}\right)$$

The input spike times is represented by t^*f*^ and δ(*x*) is a dirac delta function which follows


(6) $$\delta \left(x\right)=\{\begin{array}{c} 1\;ifx=0\\ 0otherwise \end{array}$$

The same mathematical formulation was applied to the Purkinje layer Eq. (7)


(7) $$PSP_{PC}=w^{T}x_{Grc}\left(t\right)=\sum w_{Grc}x_{Grc}\left(t\right)$$

During adaptation and learning, each of the spike train sequences were convolved with a kernel function Eq. (8)–(9)


(8) $${\tilde{x}}_{MF}\left(t\right)=\sum_{t_{MF}^{f}\in F_{MF}}\kappa \left(t-t_{MF}^{f}\right)$$

(9) $${\tilde{x}}_{Grc}\left(t\right)=\sum_{t_{Grc}^{f}\in F_{Grc}}\kappa \left(t-t_{Grc}^{f}\right)$$

Where *F*_*MF*_ and *F*_*GrC*_ were the set of spike trains inputs to the respective granular and Purkinje cell layers.

#### Decoding spiking information for classification

A decoder block follows the spiking cerebellar network to decode the firing behavior to real-world information. The decoding network consisted of an input layer with nodes based on the time bin, a hidden layer with two nodes and an output node. The output was decoded using rate-coded information from the Purkinje cell spike patterns and a convoluted neural network employing a SoftMax classifier (Algorithm 3). Rate coding was performed by dividing the time interval into time bins of 50 ms and counting the number of spikes in each bin. Spike count was stored as a vector and was used as the input layer of the convolution neural network. Values for all nodes were computed using a weighted sum of inputs.

**Algorithm 3:** Decoding using a rate coded convoluted network.

1. For each output feature **y** 2. Divide the time interval **T** into **B** time bins of time **t** : T = B*t 3. Count the number of spikes in each bin as vector **C_s_** = **{n_1_, n_2_….n_B_}** 4. Assign **[C_s_]** set as input layer and No. nodes ***j = length*(C_s_)** 5. Assign No. Hidden layers ***i = 1***; and No. hidden nodes ***j = 2*** 6. Assign No. of output node ***j = 1*** 7. Assign weights ***w*** were set at each layer of size j * i 8. For each layer ***i*** 9. For each node ***j*** 10. Compute **s_j_** = ∑**w**_**j,i**_**x_i_** + **b_i_** 11. Compute $\text{f}\left({\text{s}}_{j}\right)=\frac{1}{1+e^{-s_{j}}}$ 12. End for 13. End for 14. Compute output node **s** = ∑w*x + **b** 15. Compute $\text{f}\left(s\right)=\frac{1}{1+e^{-s}}$ 16. Check if **f**(**s**) ≤ **0.5** 17. Assign ***y’ = 1*** 18. Else 19. Assign ***y’ = 0*** 20. End if 21. End for

#### Learning rules and adaptation

Optimization of the network was done by correcting the network connection weights with calculated errors. The predicted output was compared with the actual to compute errors. During the training phase, connection weights were updated at the granular layer (**w**_*MF*–*GrC*_), Purkinje layer (**w**_*GrC*–*PC*_), and the decoding network layer (Algorithm 4).

**Algorithm 4:** Learnadapt for network learning and weight adaptation.

1. Compute the error ***e = y-y’*** Where **y** is the actual output and **y’** is the predicted output 2. Compute the adapted weight for **w_MF–GrC_** 3. For each **MF** 4. For each **GrC** 5. Assign learning rate ***l_*r*_ = 0.1*** 6. Compute **w_MF–GrC_(n+1) = w_MF–GrC_(n)** -(l_r_*e* w_MF–GrC_(n)) 7. End for 8. End for 9. Compute the adapted weight for **w_GrC–PC_** 10. For each **GrC** 11. For each **PC** 12. Assign **l_r_ = 0.35** 13. Compute **w_GrC–PC_(n+1) = w_GrC–PC_(n)** -(l_r_*e*w_GrC–PC_(n)) 14. End for 15. End for 16. Compute adapted weight at Rate Coded Convoluted network 17. Compute error signal e_*s*_ = (y-y’)*y’*(1-y’) 18. Assign ***l_*r*_ = 0.15*** 19. Compute *w*(n+1) = *w*(n)-(l_r_*e_s_*s(n)) 20. For each hidden layer ***i*** 21. For each hidden node ***j*** 22. Assign ***y’ = s_*j*_*** 23. For each previous layer node k 24. Compute △**e** = ∑_**es_j_**_(**i**+**1**)*w(**i**)_**jk**_ 25. End for 26. Compute _**es_j_**_(**i**) = △e*y′*(**1**-**y**′) 27. Check if i = 1 28. Compute *w*_*ji*_(n+1) = *w*_*ji*_(n)-(l_r_*e_s_*x_*j*_(n)) 29. Else 30. Compute *w*_*ji*_(n+1) = *w*_*ji*_(n)-(l_r_*e_s_*s_*j*–1_(n)) 31. End for 32. End for

The learning rule for updating synaptic weights was employed as in Eqs (10)–(12)


(10) $$\Delta w_{Grc}\;(t)=\lambda {\tilde{x}}_{Grc}\;(t)\;e_{m}$$

(11) $$\Delta w_{MF}\;(t)=\lambda {\tilde{x}}_{MF}\;(t)\;e_{s}$$

Where λ was the learning rate, *e*_*s*_ and *e_m_* were the sensory error and motor error, respectively, used for the prediction task. For the classification task, a single error representation was used (*e*_*s*_ and *e*_*m*_ errors were considered the same) and was calculated using the general form,


(12) $$e\left(t\right)=\left(y_{d}\left(t\right)-y_{out}\left(t\right)\right)$$

Here, *y_d_* was the desired motor or sensory commands and *y*_*out*_ was the predicted.

The weights across the network were modified (10.11) based on the architecture of the modeled network and were critical to the changes observed in the input layer of the model as observed in deep convolution networks (Diehl et al., 2015; Pfeiffer and Pfeil, 2018). Only a small portion of the granule cells was turned silent, and this sudden sparseness itself was important for the learning process (Galliano et al., 2013). Bidirectional plasticity was introduced at both the synapses, with the weight at MF-GrC and GrC-PC being updated based on a Hebbian-like learning rule (error instead of inputs), and weights at the decoding network were updated based on a modified backpropagation learning rule. Optimized learning rates were used for the study, which was obtained from an extensive trial-and-error method (see Supplementary Method 3) based on the rate of cerebellar learning at various synapses, with PF-PC having the fastest learning to the PC-DCN with the slowest learning (Schweighofer and Arbib, 1998; D’Angelo et al., 2016b; Herzfeld et al., 2020). For the cerebellar network, the learning rates for the algorithm were kept as low as 0.1, 0.35, and 0.15 [optimal choice based on trial-and-error (see Supplementary Method 3 and Supplementary Figure 3)] for the MF-GrC, PF-PC, and PC-DCN connections, respectively.

The MATLAB (Mathworks, USA) source code for the cerebellar network for classification is available at https://github.com/compneuro/DLCISNN and will be made available on ModelDB.

### Dataset

To train and validate this model (Algorithm 5), various datasets from the UCI repository (Lichman, 2013) were used. The algorithm was tested on autism spectrum disorder (ASD) for children, adolescents and adults dataset (Thabtah, 2017), Iris dataset, play tennis (Soman et al., 2005), and robotic arm dataset (Vijayan et al., 2013). All the 3 ASD datasets had 19 features with more than 100 instances, iris and play tennis had 4 features, and the robotic arm dataset had 9 features. WEKA data mining platform (Witten et al., 2011) and the deep cognition platform (deepcognition.ai) were used for comparing the cerebellum model with other ML algorithms.

**Algorithm 5:** CISNN training and testing.

1. Get the dataset **D_T_** from the user 2. Split **D_T_** into training (**D_Tr_**) and test data (**D_Ts_**) using 66% split 3. For each **D_Tr_** 4. Encoding the realworld data **T_mfspiketime_** = **MF-Granular input** **Encoding (D_Tr_)** 5. Create spiking neural network **T_pcspiketime_** = **Cerebellum Inspired** **Spiking Neural Network (T_mfspiketime_)** 6. Decode the spiking information to output **y’ = Decoding (T_pcspiketime_)** 7. Compute the error ***e = y-y’*** 8. End for 9. For each epoch 10. Update network weights using **Learnadapt(e)** 11. End for 12. For each **D_Ts_** 13. Encoding the realworld data **T_mfspiketime_** = **MF-Granular input** **Encoding (D_Ts_)** 14. Create spiking neural network **T_pcspiketime_** = **Cerebellum Inspired** **Spiking Neural Network (T_mfspiketime_)** 15. Decode the spiking information to output **y’ = Decoding (T_pcspiketime_)** 16. End for

### Modeling motor articulation control

A modified version of the cerebellum-inspired spiking neural network (Vijayan et al., 2017; Figure 2) was also used to predict the inverse kinematics of a simple sensor-free robotic articulator. However, inverse kinematics was ill-posed concerning the solution’s existence, uniqueness, and stability, and solving this required multilayer networks with different regularization functions (Ogawa and Kanada, 2010). The current model was designed to solve these problems. The model input features were the kinematic parameters which were end-effector coordinates or motor values or both, that were provided to the network model to predict the output in the form of class labels or motor angles. Both input and output were decided based on the task. A 6-degree-of-freedom (DOF) robotic arm dataset generated from the robotic arm was used to test the predicted result (Nutakki et al., 2016; also see Supplementary Figure 1). In this case, the cerebellar network model for prediction consisted of 21 MF, 279 GrC, 1 GoC, and 6 PC for encoding seven features per neuron. The neurons were scaled up for different tasks based on the input features and user-defined MF count (see Supplementary Method 5 and Supplementary Table 1). As the cerebellum is known for error-driven motor learning, a dual error representation (sensory error and motor error) was considered for the error correction (Popa et al., 2016). Decoded values were used to calculate the two types of errors: sensory error and motor error. The motor error was considered as the difference between the desired and the predicted motor angle, while sensory error represented the change in end-effector coordinates defined in the Cartesian space. A geometric estimate was used to calculate the end-effector coordinates from predicted motor angles, and this change in end-effector coordinates was considered as a sensory error. The sensory error feedback was to minimize the error, which biologically would have been done by the other loops in the circuit (Doya, 2000). In the case of motor error, it represents the error signal through the CF to the PC. Learning at the different layers has been designed based on the two types of errors. The sensory error was used to update the weight at the MF-GrC and the motor error at the GrC-PC and the layers in the decoder. Normalization functions were used to optimize the predicted joint angles, which was considered a regularization function. The predicted values were normalized using a min-max normalization function from a 0–1 range to 0–4.45, which corresponded to the angle in radians.

### Sparseness and capacity measures

The sparseness index (SI) (Tartaglia et al., 2015) of the granular layer cells was calculated to measure the sparseness involved in the coding scheme at the granular layer. The SI was calculated as in Eq. (13).


(13) $$SI=\frac{1-A}{1-\frac{1}{n}}$$

Where A was calculated as in Eq. (14) and n is the number of stimuli.


(14) A=(∑1nvi/n)2∑1n(vi2/n)

Where *v_i_* represents the mean firing rates to a set of stimuli, and SI can take only values between 0 and 1.


(15) $$SI=\left\{ \begin{array}{c} 0\;whenv_{i}=vforalli\;\left(A=1\right)\\ 1\;whenv_{i}=vandv_{j\neq i}=0\;\left(A=\frac{1}{n}\right) \end{array}\right.$$

Analytically, it was difficult to estimate the number of input-output associations that can be stored in a neuron as it depended on the weights’ dimensionality and the number of labeled patterns. Extensive computer simulations were required to calculate the number of input-output associations stored in a neuron (Memmesheimer et al., 2014). The storage capacity (α_*c*_) of the Purkinje cells (Rubin et al., 2010; Clopath et al., 2012; Vijayan et al., 2015) in the network was also calculated to quantify the number of input-output associations that can be stored. The storage capacity with respect to stability constant (κ) and mean rate of output spike time (r_*out*_ τ) was calculated Eqs. (17) (18) based on the duration of input pattern (T) for both the granule cell and Purkinje cell and the number of active MFs (N_τ_) in a particular time window. The stability constant of the neuron was estimated using the Eq. (16) (Rubin et al., 2010), where τ_s_ and τ_m_ represent the synaptic time constant and membrane time constant.


(16) $$\kappa =\frac{T}{\surd \tau_{s}\tau_{m}}\;$$

(17) $$\alpha_{c}=\frac{lnln\kappa }{2ln2}$$

(18) $$\frac{T}{N_{\tau }}=\alpha_{c}\left(r_{out}\tau \right)$$

The membrane time constant (τ_*m*_) for the Purkinje cell was taken as 64 ± 17 ms (Roth and Hausser, 2001) and the granule cell was taken as 1.4 ± 0.12 ms (Delvendahl et al., 2015). The synaptic time constant (τ_*s*_) was calculated as τ_*m*_/4 (Rubin et al., 2010). When MF inputs were provided, the granule cells had a r_*out*_ in the range 10–50 Hz (Gabbiani et al., 1994; D’Angelo et al., 2001). In the case of PC, which was a spontaneous firing neuron model has a r_out_ of 30 Hz, and as PF impinged onto the PC dendrites, the r_out_ had a range of 30–500 Hz (Khaliq and Raman, 2005; Masoli et al., 2015). For the theoretical calculation of storage capacity, two inequalities exist; in the case of GrC, the r_out_τ < = 0.1 was used and for PC r_out_τ > = 1.

### Graphics processing unit implementation of the cerebellar input layer model

To compare the computational costs during scaling, a modified version of the network implementation on the GPU platform was employed (see Supplementary Figure 2). Several network modules resembling the cerebellar microzones were reconstructed by maintaining the realistic neural density as reported in Solinas et al. (2010). Since granule neurons were more numerous than the other types of neurons reconstructed in the cerebellar cortex and the computations in the granular layer were embarrassingly parallel, GPU was used as a candidate to parallelly execute the cerebellar granular layer neurons. For GPGPU simulations, data-parallel processing mapped data elements to parallel processing threads, and the memory requirements for GPU processes were calculated at runtime, suggesting our model was automatically scalable in terms of both computational units and memory requirements (Nair et al., 2014).

## Results

### Cerebellar circuit operates as a deep learning neuronal machine

The encoding algorithm attributed spike timing and precision as a data-dependent coding feature. Reliability of input transformation has been associated with the efficacy of spike generation attributed to MF-GrC relays as well as spike precision and timing (Cathala et al., 2003). The initial convolution layers represented transformation by the granular layer followed by aggregations at the Purkinje layer representing the bidirectional plasticity modulated by PF-PC input convergence (Jörntell and Hansel, 2006). For classification and trajectory prediction-based studies, the number of spiking neurons in the input layer and the convolution layer increased with an increase in the number of features. Each feature set was encoded as an input pattern of spike trains. The number of MF was chosen at the user’s discretion, and in the current model, seven neurons were used as they could represent the center-surround spread of data. The input data were mapped to a higher hyperspace allowing seven different sets of patterns for each feature in an instance, suggesting the cerebellum input layer mapping modalities involve input transformations like those reported for sensory tasks, such as VOR and EBCC (Medina and Mauk, 2000).

**FIGURE 3:**
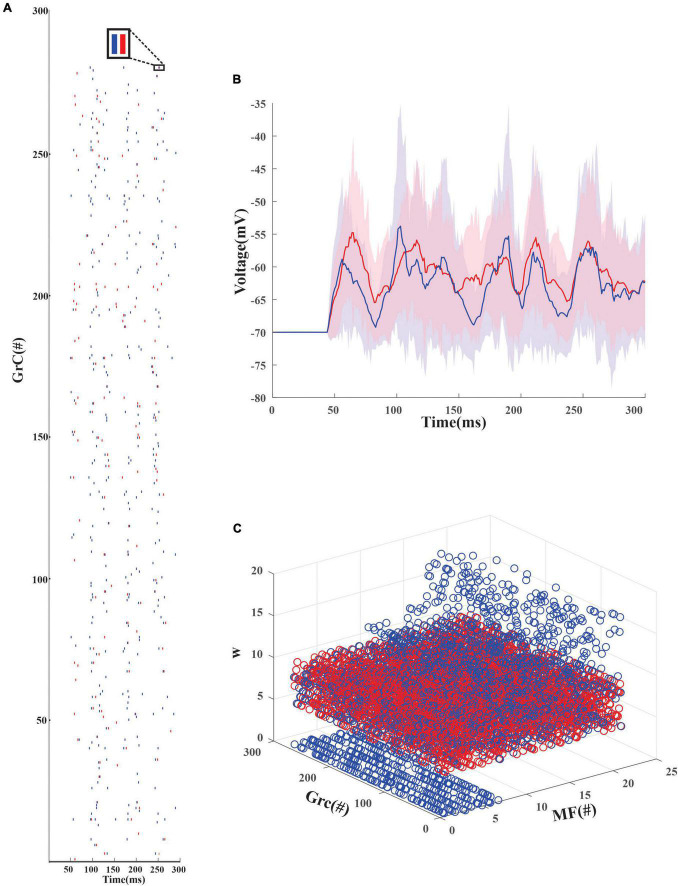
Firing rate distribution encoded input data in the modeled granule cells in the network. **(A)** Firing dynamics of GrCs pre and post-learning for 300 ms show the spread of temporal data in the input granular layer resulting from a combination of 4 random MF connections. The red dots represent the spike times of 279 GrC pre-learning and the blue dots represent the updated spike times post-learning. The inset shows the change in the firing dynamics at a particular time point for 1 GrC. Post-learning, some of the granule cells which were firing (e.g., only red raster in GrC1) turn quiescent. **(B)** Average voltage traces of granule neurons with standard deviation showed minimal change in the firing dynamics before (red) and after (blue) learning **(C)**. Weights before (red) and after (blue) learning suggest, post learning some GrCs adapt the weights to remain in a state of non-firing.

An inhibitory Golgi behavior was modeled to provide inhibitory input to the network, and it was observed that when an inhibitory spike train was introduced to the network, the granular layer showed a synchronous behavior (Figure 3A). From the average firing rate of the GrCs, it was also observed that minimal learning happens at the MF-GrC (Figures 3B,C) when compared with the PF-PC synapse. After learning, it was also observed that some of the granule cells turned silent (Figure 3A). There were also cases wherein after the initial iteration there were granule cells that were in a quiescent state and as learning progresses these neurons tend to fire and other neurons tend to change to a quiescent state. Learning at the different layers was representative of the plasticity-based learning at the MF-GrC, PF-PC, and PC-DCN. PC-DCN in the model was a transformation function that has error-based learning. It was observed that for a classification-related study when the learning rate was increased from 0.01 to 0.23 at the PC-DCN synaptic model, the training was fine-tuned at less than 5 iterations, thus mimicking the cerebellar fine-tuning behavior.

### Cerebellum-based model noisily encodes input patterns

**FIGURE 4:**
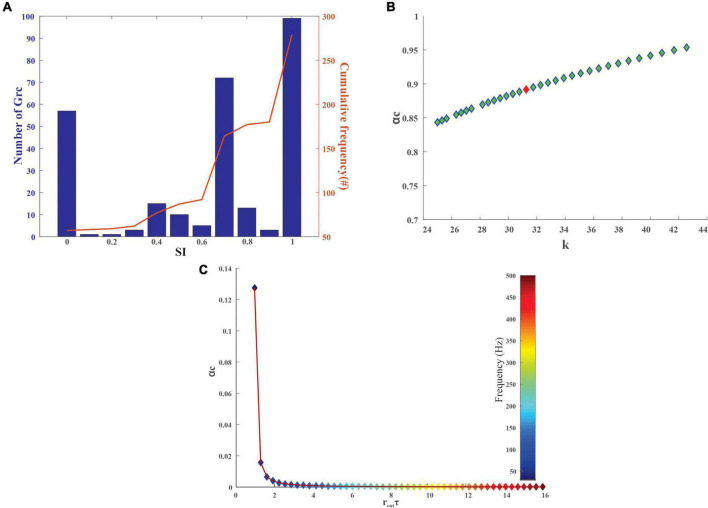
Quantification of stored information in the granule layer neurons of the cerebellum-inspired model. **(A)** Granular layer encoding relied on connection sparseness. The sparseness index of 279 granule neurons in the network with the encoded stimuli as input was calculated after 50 epochs. As iterations increase the granule cells tend to be sparser (SI nearing 1) as seen from the cumulative frequency. **(B)** Storage capacity (α_c_) of granule neurons with respect to stability constant (κ) represents the formation and storage of new patterns. The red dot represents α_c_ for a GrC whose membrane time constant (τ_m_) was 1.4 ± 0.12 ms. **(C)** Storage capacity (α_c_) of GrC with a firing frequency of 10–50 Hz was calculated and as the mean firing output spike time (r_out_τ) increases, the storage capacity decreases steeply suggesting that a reduction in firing frequency leads to an increase in pattern storage.

The degree of sparseness for granule layers neurons was analyzed using the SI measure. The SI depended on the firing rate based on the stimulus, and the mean SI for the granular layer neurons was observed to be 0.9855 ± 0.0625 before the training and 0.6276 ± 0.22 after the training (Figure 4A). As the SI increased, the signal-to-noise ratio increased, thus increasing the memory capacity, thereby decreasing classification error (Babadi and Sompolinsky, 2014; Rössert et al., 2015).

**FIGURE 5:**
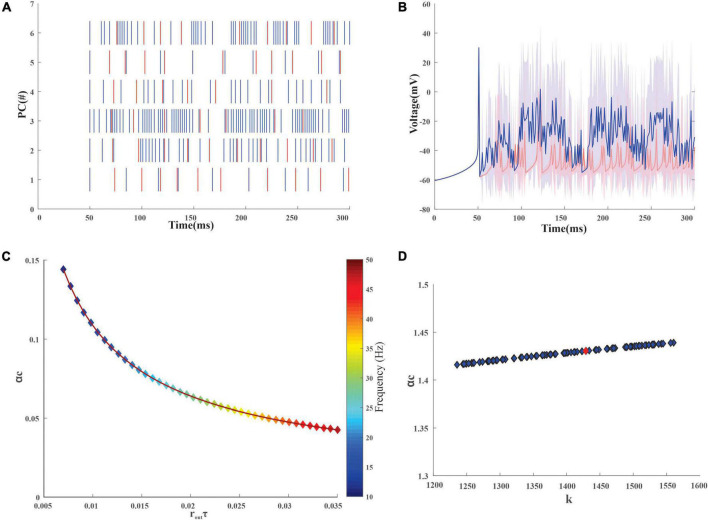
Firing dynamics and quantification of stored input-output association in the Purkinje layer neurons. **(A)** Spike variability of the 6 Purkinje neurons in the network, pre (red) and post (blue) learning. As learning adaptation improves, the firing frequency of the PC was also observed to increase. **(B)** Superimposition of the average firing dynamics of PC (pre and post) showed an increase in the firing frequency after learning adaptation with an increase in standard deviation **(C)**. Storage capacity (α_c_) of PC with a firing frequency of 30–500 Hz was calculated and as mean firing output spike time (r_out_τ) increases, the storage capacity decreases gradually **(D)**. Storage capacity (α_c_) of PC with stability constant (κ) representing the storage of new patterns at the synapse. Purkinje neuron (red dot) with a membrane time constant (τ_m_) of 64 ± 17 ms was used for the reconstruction of the network.

After the training, the PC spikes increase and tend to increase the firing average also (Figures 5A,B). The storage capacity (or the length of sequence that could be learned) of the modeled neurons was analyzed to understand the neurons’ ability to store stimuli-response associations. The mean output spike times for the PC range from 30 to 500 Hz (Khaliq and Raman, 2005; Masoli et al., 2015) and with an increase in firing frequency, storage capacity decreased (Figure 5C). For the granule cells whose mean output spike times range from 10 to 50 Hz (Gabbiani et al., 1994; D’Angelo et al., 2001), the capacity of the cell was found to reduce with an increase in the firing frequency (Figure 4C). The reliability of the neuronal models was calculated from the storage capacity (Figures 4B, 5D). As the reliability factor (k) increased, the firing frequency also increased without increasing the information capacity of the neuron. It was observed that the PC showed a higher storage capacity compared with the GrC. The PC was attributed to an increase in storage capacity (Varshney et al., 2006). A comparison study (Vijayan et al., 2015) was previously carried out on the storage capacity estimated and indicated a similar result of ∼0.2–0.3.

### Comparison with other machine learning algorithms

**FIGURE 6:**
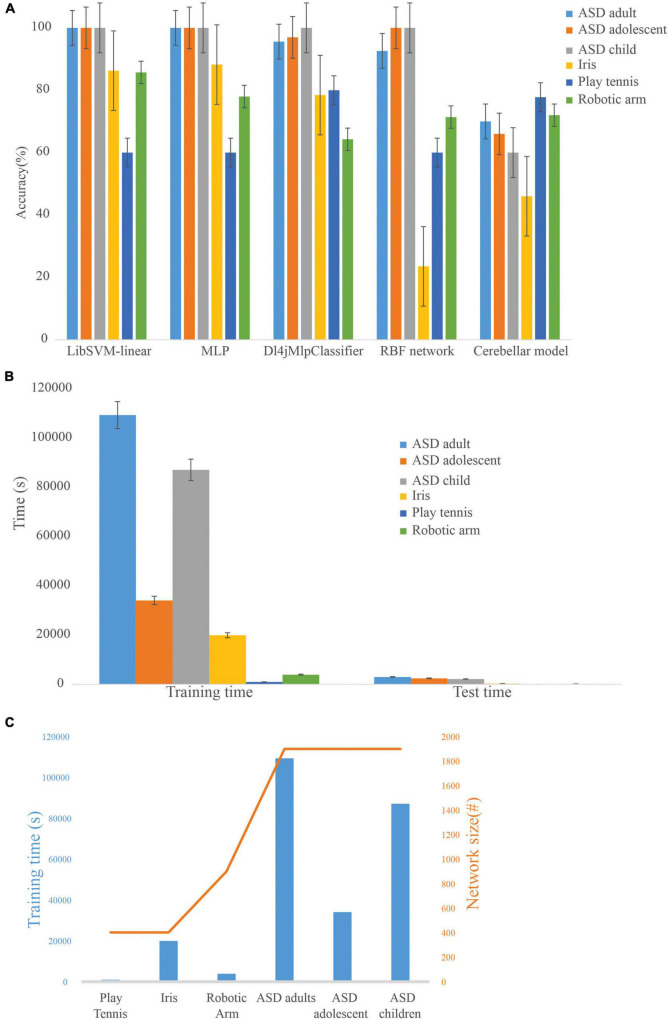
Comparison of the cerebellar model with machine learning algorithms on standard datasets **(A)**. Training accuracy of machine learning algorithms on different datasets showed that the cerebellar model could be used on a flexible dataset and rote learning was minimized. **(B)** Computational time required by the cerebellar model during the training and testing phase on various datasets. **(C)** Dependency of network size on training time.

ML algorithms, LibSVM, MLP classifier, Dl4jMlp classifier, and RBF network were used to compare the performance of the cerebellum-inspired neural network model (Figure 6A). The cerebellar network showed an increase in accuracy with training epochs when the learning rate at PC-DCN was less than 0.01 and saturated slowly compared with the other ML methods. It was also observed that as the learning rate at PC-DCN increases from 0.01 to 0.23 accuracy reaches optimum by less than 5 epochs which is one of the features of DLNs. Although less efficient on small datasets, deep learning algorithms show 100% accuracy, which may be attributed to rote learning. Among the classifiers, the Dl4jMlpClassifier, which resembled the cerebellar model had a higher test efficiency of 96.96% for ASD_adolescent dataset, 95.6% for ASD_adult dataset, 100% for ASD_adult dataset, 78.4% for Iris dataset, 80% for play_tennis, and 64.28% for robotic arm data.

### Feature generalization capability was input dependent

The model was used to evaluate the classification accuracy on different ML datasets using a 66% split. To test the generalization capabilities of the cerebellum-like spiking network model, various ML datasets were used, and the number of instances involved in the training process was: 131 (ASD_adult), 65 (ASD_adolescent), 164 (ASD_child), 99 (Iris), 27 (robotic arm), and 10 (play_tennis). Training time and test time increased with an increase in the number of features as the network formed was based on the number of input features (Figures 6B,C). Since input organization was not strictly connected to a layered set of neurons, the complexity of a feature in relation to all other features seemed more relevant while recruiting a dataset-driven architecture. Scaling to very large datasets, required parallelization of the inputs layer geometry reducing the time complexity associated with the network. In this direction, we attempted a simpler input code as volume geometry without adaptation (Supplementary Figure 2). With the granular layer computations being inherently parallel, we compared the implementation efficacy of 3.5 million granule neurons (Supplementary Figure 2). Large-scale implementations were feasible on GPGPUs with code parallelization. A smaller network model was utilized to compare CPU and GPU implementations, indicating multi-factor speedup with the GPU version (Nair et al., 2015). The generalization may also be attributed to a subset of dense neurons being activated, weights adapted, and sparse recoding with a larger number of neurons in physiologically feasible geometries (Diwakar et al., 2011).

### Motor articulation control with the cerebellum-inspired spiking neural network model

**FIGURE 7:**
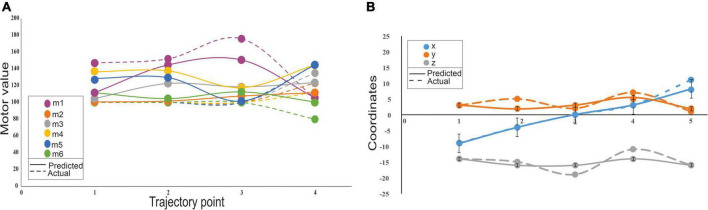
Variability on sensory and motor prediction for a 4-point trajectory by a low-cost arm. **(A)** Motor variability: Variability of the motor predictions was carried out using the cerebellar model that predicts the motor consequences from sensory commands. Motor errors were calculated from the actual values (dotted lines) and predicted (solid lines) for 6 motors. **(B)** Sensory variability: Sensory consequences of the predicted motor values were calculated using forward kinematics and errors associated were calculated as the difference from the actual to the predicted end-effector coordinates. Sensory error representations showed minimal variations and represented in the error bars some of which were negligibly small and very insignificant to be displayed.

Cerebellum-inspired SNN could also predict trajectories by generating motor angles from end-effector coordinates without explicit kinematic representations. The network model represented the inverse kinematics transformations and predicted trajectories. For a single data point, the sensory consequence represented by the 3 end-effector coordinates (such as X, Y, and Z) was used to predict the 6 theta values, with the use of an encoder unit, 307 neurons, and a decoder for motor values, which converted to a “motor command” for the next point. For a short trajectory with four points (Figure 7), 1,228 neurons were needed for the robotic arm to trace the path. It was observed that the motor values changed more (Figure 7A) when compared with the end-effector coordinates, with negligible errors (Figure 7B). Except for the first motor value, all others were initially identical and had changed only in the third and fourth points, so the dotted line appeared to be superimposed (Figure 7A). The motor error and sensory error calculation were based on the difference between predicted and desired motor angle/end-effector coordinates. The motor error was used to update the weights at PF-PC and PC-DCN weights, and sensory error was used to update the weights at the MF-GrC. After training the network, a trajectory was plotted, and the change in features and output values showed minimal error suggesting the algorithm minimized both the sensory and motor error (see Supplementary Figure 4). With network configuration that matched the cerebellum’s cytoarchitecture and connectivity, the outputs matched our previous implementation (Vijayan et al., 2017). The model mimicked the feedback controller functioning like the olivo-cerebellar circuit where the PC provided negative feedback to IO through the DCN, thereby reducing the network errors.

## Discussion

The cerebellar spiking model was an abstraction of a bio-inspired architecture and could reconstruct the cerebellar network to predict and classify multimodal input. The model reduced multidimensional data using spatiotemporal characteristics of its spiking neurons and employed plasticity rules of the neuronal circuit. The model also suggests that the cerebellar microzone functions may have strong correlations to data encoding properties and may show adaptations while fine-tuning movement tasks. The current algorithm suggests granular layer geometries may help concurrent and implicit executions of large-scale pattern recoding problems, as shown by the model’s feature classification.

### Pattern classification depended on granule neuron encoding of the stimuli, purkinje cells temporal dynamics, and induced plasticity

MF inputs allow pattern representation through an abundance of the granule cells engaging the modalities of sensory patterns by similar encoding mechanisms. The network model used target representation to discriminate patterns from inputs. Multimodal conveyance of sensory signals through MF and the error signals by CF suggested by the Marr–Albus–Ito theory may be an efficient representation for the cerebellum-inspired classifiers to discriminate specific patterns using the feed-forward and feedback mechanisms of the granular layer.

The algorithm employed time windowing to recode patterns, although in this current case study, only a small subset of different unique patterns was sufficient for the datasets used. In the cerebellum, GrC recoding uses the phenomena of time windowing (D’Angelo and Zeeuw, 2008), and the activation patterns in this one-to-many mapping parallel processing capabilities. The simulations suggest that time windows allow incorporating the parallel convolutional processing capability into the encoding performed by the granule cells, suggesting a specific convergence–divergence geometry could encode multiple pattern abstractions. A similar subset of GrCs may handle a different kind of task based on the same time window. GoC-GrC inhibition was crucial for pattern discrimination at the granule cell and supported the experimental data related to the time window hypothesis (Solinas et al., 2010). Inhibition-induced synchronicity could be relevant for pattern identification in the PC.

Reversible learning attributed to bidirectional plasticity at the MF-GrC and PF-PC synapses may be simplified as in the implemented model for recurrent learning tasks. A key role of such implementations would be a generalization task as reported in recent studies, and the current implementation employs the generalization to sparse and dense computations predicted by local field potential reconstructions (Diwakar et al., 2011). The model’s generalization ability depended on the overlap or intersection of activated neurons in the granule layer. Like the learning capacity of a biological organism in learning from one experience to another, feature generalization looks at the stored neural responses and uses the same responses to mimic similar input patterns. A weight change is the main difference between connections. In this current study, the SI was used to measure the feature generalization capability at the granule layer as the input data were encoded to MFs. Figure 4A shows that some neurons respond to one type of input and some do not, but most neurons follow a normal distribution representing the generalization capability. The model network can be extended to a large number of clusters of GrC (randomness can be replaced with more biologically relevant jitter) to compare experimental patterns and their ensemble manifestations.

### The cerebellar network model implicitly reconstructed inverse kinematics of a 6-degree of freedom robotic arm

A cluster of neurons may encode the position into a matching transformation representing the force position matrix. In the model, the kinematic transformation depends on the granular layer circuit and the consequential PC output by involving transformations of the network’s spatiotemporal domain and space-force geometry. In the CISNN representation, a single joint was characterized by a collection of neurons attributing to the concept of different body muscles being controlled by different lobules in the cerebellar cortex (Bolk, 1906) and hence used the same to scale up the models based on the trajectory for better performance. By disabling certain GrCs during simulations, it was observed that errors in trajectory tracking increased, and the robotic arm could not complete the desired trajectory mapping. ANN-based single-layered architecture with a feed-forward granular layer (Bratby et al., 2016) or a feed-forward Purkinje layer (Clopath and Brunel, 2013) have been previously used for pattern separation or classification. Still, an SNN with multiple layers would be better for mathematically independent tasks. The somatotopic organization of the cerebellum at a functional level may involve similar representations by the clusters of granule neurons at the input layer, and modeling-based information processing relies on the connection geometries and the afferent pathways that bring in the information. The CISNN employs the coding aspects of the cerebellum’s input layer and the discriminability of the Purkinje layer for performing sensory guidance of movement.

### Quantitative measure of the memory representation

A subset of seven unique recoded MF patterns for each input feature contributed to the distributed representation of the granule neurons in the network. A random combination of the different stimuli causes the average granule layer responses to be uncorrelated and more identical or remain in a quiescent state. But as the inhibitory neurons are introduced to the granular layer, the sparseness increases, causing variability in the neuronal connections. Changes in weight and membrane time constant were some of the other factors that affect the sparseness, which was not considered in the study. Storage of random input-output associations in the Purkinje cell was estimated using storage capacity. Compared with the granule cells, the Purkinje neuron had a higher storage capacity which could be attributed to the bistable nature of the cell and its large dendritic arbor and that memory consolidation happened at the dendritic synapses. Studies (Li et al., 2022) had shown that higher information processing capacity led to lower neuronal activity and faster responses, suggesting that during the learning phase, neural engagement increased abruptly at the start of learning and then gradually declined. For GrCs, the storage capacity reduced steeply with firing frequency proposing the role of associative mapping over the storage of input-output associations and was associated with the Purkinje cells in real circuits. As the mean firing rate increased, the storage capacity decreased gradually, as observed in studies (Memmesheimer et al., 2014) denoting an inverse relation between r_out_ and storage capacity.

### Cerebellar network model as a deep learning algorithm

Cerebellum input divergence and PF-PC mapping may serve as the convolutive coding layers employed by other DLN. A typical DLN involved multiple convolution layers, which may be the function of classification-related microcircuits in the cerebellum. In the CISNN, these convolution layers were represented by MF-GrC, MF-GoC-GrC, and GrC-PC transformations and their learning rules (Jörntell and Hansel, 2006) and match experimental data of the neurons performing temporal and combinatorial operations (Mapelli et al., 2010; Gilmer and Person, 2017). Taking advantage of these dynamics in ANN may help concurrent and implicit discriminations of large-scale patterns. From an algorithmic standpoint, the cerebellum-inspired deep learning model is scalable based on the input features and user-defined MFs and can accommodate multimodal input patterns functioning as a high dimensional coder for spatiotemporal data. Projected spatiotemporal coding properties of the cerebellum allowed converting real-world data into spike trains and were used to classify the dataset. Comparing the cerebellar model with different datasets and other standard ML algorithms suggests that the model functions as a good classifier with considerable accuracy, which tends to increase with epoch when the learning rate was reduced, suggesting an efficient learning mechanism. Even though the performance of other well-known algorithms, such as MLP, Dl4jMlpClassifier, and RBF networks fractionally outperform the cerebellar model in terms of accuracy, the model was sufficient enough to do the task of classification as well as trajectory prediction for a low-cost sensor-free robotic articulator. The robotic arm was developed in the lab whose total cost was < 5$ (Vijayan et al., 2013), and a network model with fewer neurons was able to perform the specified tasks. The cerebellum-inspired spiking neural network was optimized in less than 50 epochs suggesting that employing a feed-forward cerebellum-based model could be used as a supervised classifier and predictor for big data.

### Scaling up the spiking network model and modeling cerebellar function

MF-GrC-GoC is an internal loop that monitors the input by providing back-and forth-signals at the GrC-GoC connection. Recurrent inhibitory loops play a significant role in error minimization in the cerebellum. Some loops are computationally costly and were avoided. This may need to be reconsidered for big data operations. For computational efficacy, the algorithm did not model silent or non-spiking granule cells and the optimization of learning was based directly on the abstractions of the non-silent cells. The burden on learning was optimized by the granule cell’s silent/non-silent ratio, which could be redefined using convergence–divergence.

The coordination of articulator joints may be understood from the spiking reconstruction as in CISNN, and the scalability of the cerebellar input layer clusters can be exploited to understand the cerebellar functions in the context of robotics computationally. The algorithm does not include any sensor or visual-based inputs, thereby decoupling non-motor sensory inputs from the cerebellum-based internal models. As a deep learning algorithm, the CISNN poses many challenges, such as overfitting a model to its training data.

Prediction accuracy relied on the role of dual representation of errors (such as sensory error and motor error) (Popa et al., 2012), which may be approximated in the learning process as the model generalized the prediction process. The model’s inability to discriminate sensory or motor errors could be attributed to the lack of CF-related error feedback in the hardware we employed to generate the trajectories. Even after the training phase, some of the weights remained unoptimized due to the weight assignments, which can be resolved by introducing heuristic initialization, such as Xavier’s or Kaiming’s initialization that employed ReLu, leaky ReLu, or tanh functions.

Optimizing learning rates for the different neurons was a big challenge, requiring tweaking the different parameters without changing the neuronal dynamics. Larger models yield better performance when compared with smaller models (Li et al., 2013). For scaling up the model, a larger dataset or more MFs have to be introduced, which in turn creates a need for high computation power for running the simulations. Scaled-up SNN are now used to learn spatiotemporal dynamic patterns of biosignals and map neuronal connections created during the learning process (Gholami Doborjeh et al., 2018).

Like DLN models, the CISNN architecture may require scaling optimizations, reducing the time complexity for large datasets (with the four Vs) to solve real-world problems. Scaling the model for very large datasets required parallelization of the input layer geometry, and faster learning in terms of time complexity may be attained using GPU-based processing units. The advantage of this framework is that the algorithm can be reimplemented on GPGPUs and FPGA hardware. Although we tested with smaller sizes and resolutions, these parallel GPU implementations may allow scaling this cerebellum-inspired spiking neural network for big data and streaming data classifications.

The model may be relevant to reconstruct neurological disorders mimicking programmed failure of robotic motor joints during trajectory mapping by articulators. Although we did not implement all the 16 known forms of plasticity (Gao et al., 2012; D’Angelo et al., 2016a) and reinforcement learning in the MF-GrC pathway (Wagner et al., 2017), this cerebellum-inspired implementation currently serves as a reductionist mapping between the convergence–divergence ratios of neurons and connection geometries and could act as a testbed for understanding dysfunctions. The model is relevant and can be extended for the movement-related reconstruction of inverse kinematics as well as modeling coordination-related changes.

### Comparison with other cerebellar models

The current model was reconstructed based on known microcircuitry of the cerebellum (Eccles, 1967), electrophysiological behavior (D’Angelo et al., 2001), and significant plasticity rules (Mapelli et al., 2015). Even though there are several models available that look at the different aspects of the cerebellum (Casellato et al., 2012; Garrido et al., 2013; Antonietti et al., 2015; D’Angelo et al., 2016b; Luque et al., 2016; Yamaura et al., 2020; Kuriyama et al., 2021), the present model covers certain aspects of the cerebellum while some loops are skipped. Initial models looked at only single-layered neurons (Marr, 1969; Albus, 1971) which were extended with many other layers and plasticity rules. Models such as EDLUT (Carrillo et al., 2008) are more focused on the event-driven simulation scheme based on lookup tables that would reduce the time involved with the numerical calculation. In the current model, numerical calculations are introduced to best capture the processed information and filter the information that has to be sent. For specific tasks, there are networks with cells arranged in 3D structure geometry that can be simulated with supercomputers giving it the dynamics of the cerebellum (Yamaura et al., 2020). Compared with one of the closest earlier models (Hausknecht et al., 2016) where multiple modular cerebellar circuits have been considered to train different tasks, here the current model has considered a single module of the cerebellar architecture which could reduce the complexity of the network.

The cerebellar model was made scalable based on input data with fewer cells and loops, which has a crucial role in predicting and classifying learning data. The uniqueness of the current model is the incorporation of the recoding and associative mapping properties of the cerebellar granular layer with excitatory GrC and an inhibitory GoC, pattern discrimination property at the Purkinje layer, and the interpretational application of DCN along with an encoding and decoding module which involves the transformation of real-world data to spiking information and vice versa.

## Conclusion

The functions of the cerebellum in terms of fast computations and self-organizing the mapping of input patterns are yet to be better understood. This algorithmic model is a computational step in interpreting the complex control behind pattern reorganization of the motor and other signals while exploring the cerebellar architecture as a deep learning model. While error analysis, data sparseness, optimal adaptation of weights, and the relationships to known plasticity rules remain to be implemented, this model allows generalization within the context of spiking neural architecture-based pattern classification.

This cerebellar network model would allow to explore multimodal circuit-dependent pattern discrimination and sensorimotor transformations. The proposed algorithm shows potential in exhibiting dynamical properties, such as (1) improved accuracy and prediction with learning, (2) autonomous update, (3) neuronal network scalability with distributed plasticity. Given the theoretical goals of such an implementation, this model will also be adapted and extended to include recurrent loops and adaptive reinforcement learning and can be employed on big datasets. The model can be implemented on VLSI hardware and FPGA boards because of the simpler and modular mathematical operations.

The proposed model could help understand movement-related pattern recognition, such as playing a game of chess and improve models for motor articulation control by storing as well as predicting trajectories for path optimization, such as moving a cup of water. It could be used as a prediction mechanism for pathophysiological conditions and aid in BCI and neuroprosthesis like an artificial limb can be used as a deep learning model to understand the cerebellar function and dysfunctions.

With the cerebellar information and cellular losses that occur in neurological conditions, these models can be used to fine-tune tasks involving various mathematical complexity without significantly adapting the connectivity and circuit. With that, we believe this model may have prominent usability roles for experimental neuroscientists to hypothesize and explore other functions.

## Data availability statement

Publicly available datasets were analyzed in this study. This data can be found here: https://github.com/compneuro/DLCISNN.

## Author contributions

AV and SD conceived and developed the method, analyzed the data, and wrote the manuscript. Both authors read and approved the final manuscript.

## Footnotes

1
https://github.com/compneuro/DLCISNN
