# Supplementary material for: A cerebellum inspired spiking neural network as a multi-model for pattern classification and robotic trajectory prediction
Source: Front Neurosci. 2022 Nov 28;16:909146. doi: 10.3389/fnins.2022.909146 (PMC9742384; doi:10.3389/fnins.2022.909146)
Supplement: Supplementary file 1 [file Data_Sheet_1.docx]

Supplementary Material

# Methods: Development of a microcontroller-based low-cost robotic arm

A microcontroller was programmed to generate PWM (pulse with modulation) signals with a time period of 20ms and a duty cycle varying from 1ms to 2ms to control each servo motor in a low-cost robotic arm (Vijayan et al., 2013). Microcontroller was interfaced through a serial port to the computer, where the desired motor angles were transferred to the controller (Supplementary Figure 1.). The GUI was developed in Adobe ActionScript (Adobe, USA) with MATLAB (Mathworks, USA) as backend to trigger the robotic articulator. This robotic arm was part of our remotely controlled virtual laboratories and was online until recently.


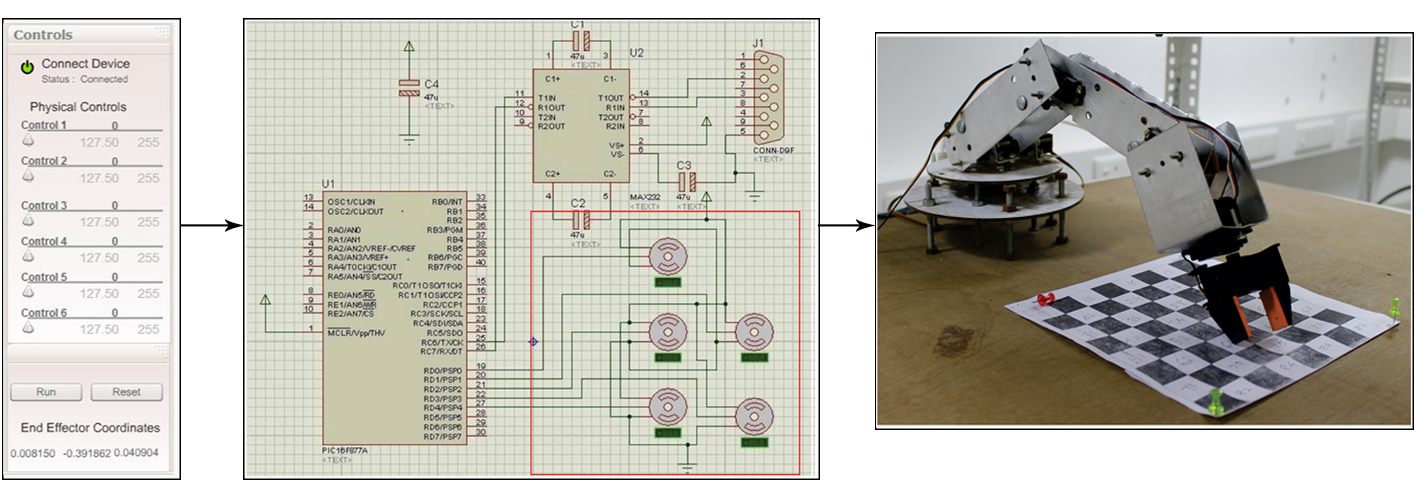


**Supplementary Figure 1. Architecture and functional process associated with the extraction of robotic arm data.** A system controllable articulator was developed as a workbench to test and validate the data extracted. The extracted data i.e., the motor values was given to the microcontroller which is then forwarded to the robotic articulator to perform the corresponding task.

# Methods: GPGPU implementation of cerebellar input layer model

A large-scale cerebellar network was reconstructed and parallel simulated using graphic processing units (Supplementary Figure 2.A.). As reported earlier, a 3D volume of the cerebellum with 100μm edge length contains granule cells with density 4 x 10^6^ / mm^3^ (Solinas et al., 2010) (Supplementary Figure 2.B). Each granule cell receives one to four excitatory connections through mossy fibers and one to four inhibitory connections through Golgi cells. Golgi cells were created to compensate for the number of inhibitory connections that should occur in the network. The number of glomeruli was calculated using the reported convergence-divergence ratio of the mossy fiber-granule cell connections. Each glomerulus includes a mean of 53 dendrites from different granule cells and each granule cell emits on an average 4 dendrites where granule cell dendrites could not reach glomeruli farther than 40µm. Only one Golgi cell axon enter a glomerulus and the same axon is not allowed to access another glomeruli which share granule cells. A maximum of 2000 granule cells were inhibited by a Golgi cell since each Golgi cell can access at most 40 glomeruli. Each Purkinje neuron received excitatory inputs from 100,000 granule neurons (Houk et al., 1996) which also received inhibitory inputs from the stellate and basket neurons. These two inter neurons received excitatory inputs from the granule neurons. DCN neuron is also simulated which carried the sole output from the cerebellum to upper motor regions. These cells were placed inside the 3D volume with uniform random distribution.


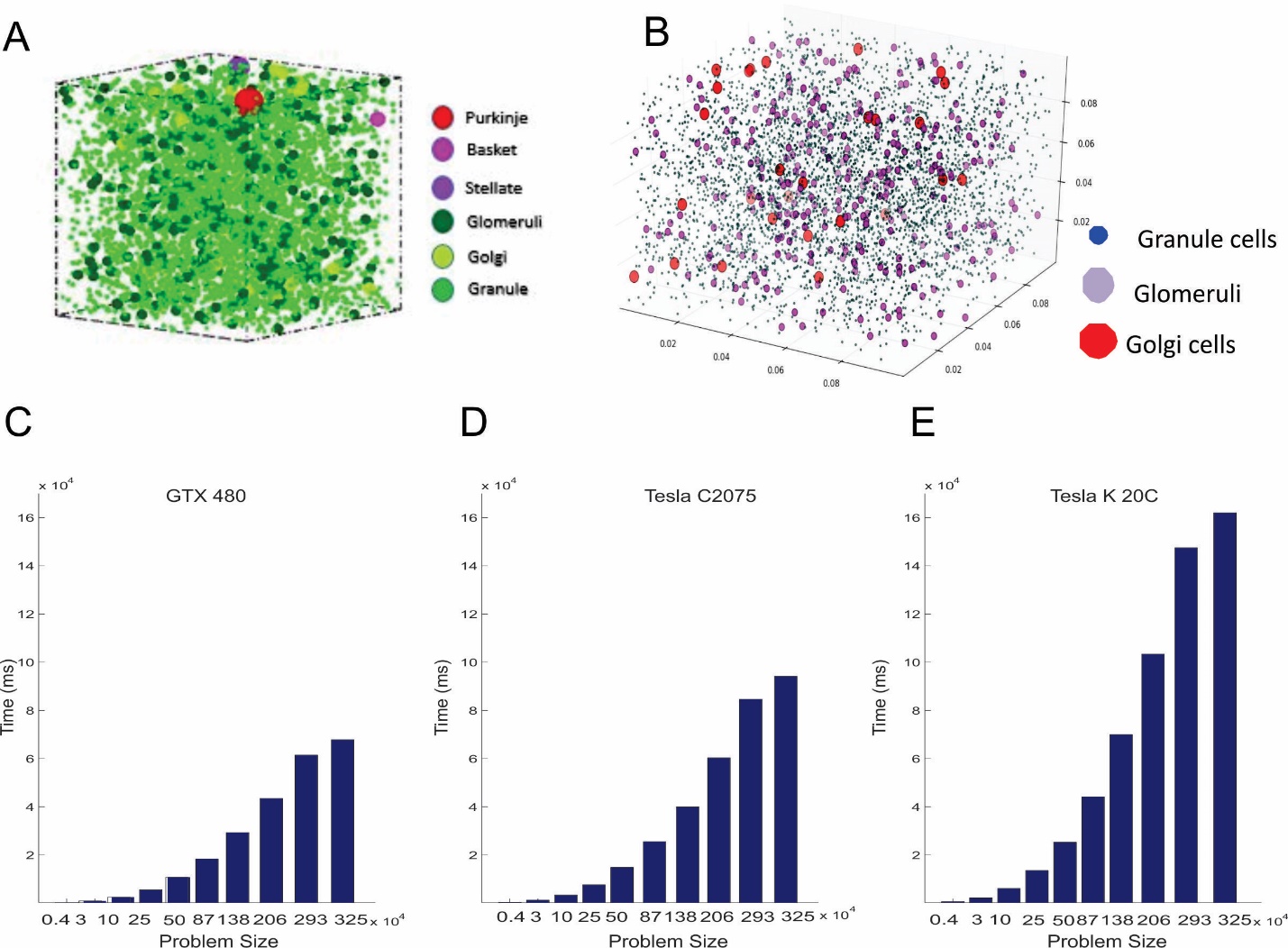


**Supplementary Figure S2 Cerebellar network reconstruction in graphic processing units. A.** Spatial distribution of various cerebellar neurons in 1mm3 volume of cerebellar tissue. **B.** Distribution of granular layer neurons modeled to be packed inside a cube with granule cell density 4x10^6^/mm^3^. Glomerulus receiving mossy fibers, granule cell dendrites and Golgi cell axon with the neurons connected through synapses using biologically estimated convergence-divergence rules. **C.** Running time versus problem size plot for large scale simulation of cerebellar granular layer neurons in different GPU cards such as GTX 480. **D.** Tesla C 2075. **E.** Tesla K20C

The cerebellar network reconstructed was both homogeneous and embarrassingly parallel. In order to achieve automatic scalability and increased efficiency, we adopted a single instruction multiple data paradigm for the simultaneous execution of different parts of the network on graphic processing units. Data-parallel processing mapped data elements to parallel processing threads. The essential sequential components of the simulation such as initialization of the inputs and simulation parameters, network construction etc. were performed in an Intel Xeon CPU with 8 cores running on 2.4 GHz clock speed. The 3D network was constructed in the CPU and the neurons and connection information were sent as 1D arrays to the NVIDIA GPU. The network of granule cells with 1 to 4 random MF connections with and without inhibition from Golgi cells were then parallel simulated in the GPU. Each neuron was mapped to one thread of execution in the parallel GPU blocks and both thread level and block level parallelism were explored. The memory requirement for GPU processes were calculated at run time (Supplementary Figure 2. C, D, E) and the number of thread blocks were allocated in a scalable manner

# Methods: Optimized learning rates based on extensive trial and error method

The learning rates were optimized for the plasticity condition manually through an extensive trial and error method (Hausknecht et al., 2016). We have explored the learning rates of the algorithm in context to the cerebellar plasticity: with PF-PC having the fastest learning (Schweighofer & Arbib, 1998) and PC-DCN the slowest (D’Angelo et al., 2016) which was also specified in a recent study (Herzfeld et al., 2020). Based on the minimal error, the learning rates were calculated from a range of values. For MF-GrC the learning rates were considered from a range 0-0.25, at PF-PC the range was 0-0.5, and PC-DCN it was 0-0.15. Optimized learning rates were 0.1, 0.35 and 0.15 for the MF-GrC, PF-PC and PC-DCN connections respectively (Supplementary Figure 3).


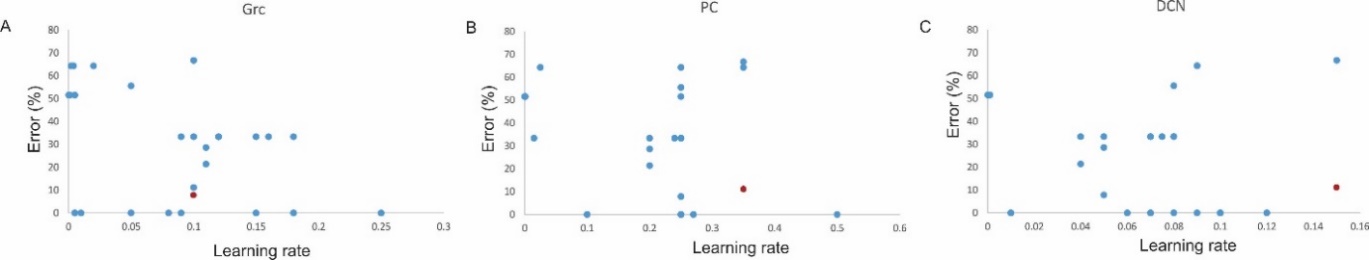


**Supplementary Figure S3 Optimization of learning rates** Error percentage of the algorithm with changing learning rate at different synapses. The red dot represents the learning rates used for the cerebellum-inspired spiking neural network. **A.** Learning rate of 0.1 for MF-GrC plasticity selected from the range 0 – 0.25. **B.** Learning rate of 0.35 for PF-PC plasticity selected from the range 0 – 0.5. **C.** Learning rate of 0.15 for PC-DCN plasticity selected from the range 0 – 0.15.

# Results: Motor error and sensory error reduced after multiple epochs

The cerebellar network was trained for a prediction task for 200 epochs and simulations followed a general trend of drop in error with iteration. In this study, 2 different errors were considered: motor error and sensory error. The motor error was calculated as the change in the predicted motor angles from the desired (Supplementary Figure 4.A) whereas the sensory error was represented by the change in end effector coordinates from desired (Supplementary Figure 4.B). The low-cost articulator that we had developed had a variability of ±3cm as reported in (Vijayan et al., 2013) Therefore, when the total sensory error fell around 5cm, the iteration for the training was terminated.


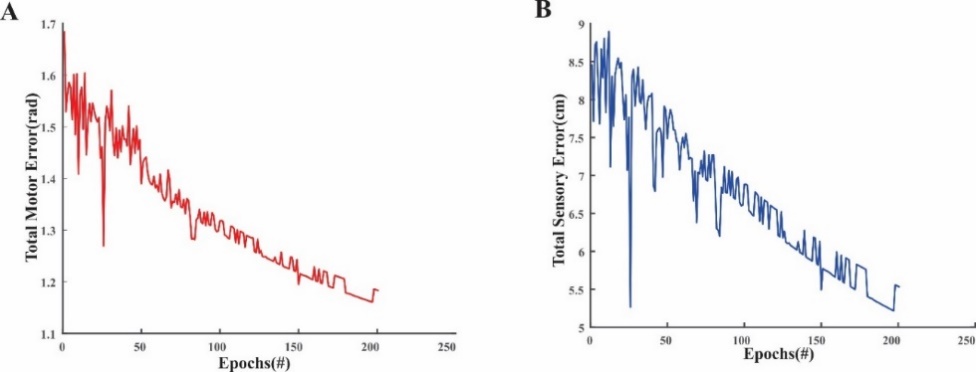


**Supplementary Figure S4: Motor and sensory errors decreased with increased iterations**. **A.** Motor error: Calculating the differences between the desired and predicted motor angles and **B.** Sensory error: Calculating the difference between the desired and predicted end effector coordinates.

# Results: Network configuration with varying input neurons and features

# The network was made scalable based on the input features and a user-defined MF count given at runtime. Features in a dataset were encoded to user-defined neurons at runtime (e.g., 4 features mapped 7 neurons each). Each feature maps to 7 neurons so totally for a 4 featured datapoint, there would be 28 input neuron (MF) which was mapped to GrC and then to PC. The output of PC was decoded to the final output through 3 layered network. For the play tennis dataset with 7 user-defined neurons, the training time was 971.86s for a network size of 401 neurons (28MF, 371GrC, 1GoC, 1PC). For the robotic arm dataset with 7 user-defined neurons, the training time was 3919.16s for a network size of 900 neurons (63MF, 835GrC, 1GoC, 1PC).

**Supplementary Table 1.** Total number of neurons in the network model with changing tasks and datasets

| **Task** | **Dataset** | **Features** | **MF** | | **GoC** | **GrC** | **PC** | **Total** |
| --- | --- | --- | --- | --- | --- | --- | --- | --- |
|  |  |  | *User-defined neurons per feature* | *MF* |  |  |  |  |
| Classification | Tennis | 4 | 7 | 28 | 1 | 371 | 1 | 401 |
|  |  | 4 | 9 | 36 | 1 | 477 | 1 | 515 |
|  | Arm | 9 | 7 | 63 | 1 | 835 | 1 | 900 |
|  |  | 9 | 5 | 45 | 1 | 597 | 1 | 644 |
|  | ASD | 19 | 7 | 133 | 1 | 1763 | 1 | 1898 |
|  |  | 19 | 5 | 95 | 1 | 1259 | 1 | 1356 |
| Trajectory Prediction | Arm | 3 | 7 | 21 | 1 | 279 | 6 | 307 |
|  |  | 3 | 5 | 15 | 1 | 199 | 6 | 221 |
|  |  | 3 | 9 | 27 | 1 | 358 | 6 | 392 |

# References

D’Angelo, E., Mapelli, L., Casellato, C., Garrido, J. A., Luque, N., Monaco, J., Prestori, F., Pedrocchi, A., & Ros, E. (2016). Distributed Circuit Plasticity: New Clues for the Cerebellar Mechanisms of Learning. *Cerebellum*, *15*(2), 139–151. https://doi.org/10.1007/s12311-015-0711-7

Hausknecht, M., Li, W. K., Mauk, M., & Stone, P. (2016). Machine Learning Capabilities of a Simulated Cerebellum. *IEEE Transactions on Neural Networks and Learning Systems*, 1–13.

Herzfeld, D. J., Hall, N. J., Tringides, M., & Lisberger, S. G. (2020). Principles of operation of a cerebellar learning circuit. *ELife*, *9*. https://doi.org/10.7554/eLife.55217

Houk, J. C., Buckingham, J. T., & Barto, A. G. (1996). Models of the cerebellum and motor learning. *Behavioral and Brain Sciences*, *19*(03), 368–383.

Schweighofer, N., & Arbib, M. A. (1998). A model of cerebellar metaplasticity. *Learning & Memory*, *4*(5), 421–428.

Vijayan, A., Medini, C., Singanamala, H., Nutakki, C., Nair, B., & Diwakar, S. (2013). Classification of Robotic Arm Movement using SVM and Naïve Bayes Classifiers. *Innovative Computing Technology (INTECH 2013),Proceedings of Third International Conference On*, 263–268.
